# Mistaken Benevolence

**Published:** 1860-04

**Authors:** 


### MISTAKEN BENEVOLENCE.

A WOMAN, with a dog on each side, hitched to a hand-cart, may be seen in any street in New-York of a bleak winter's day; or little girls with baskets, the girls, not the baskets, in tatters, filth, and rags, without bonnets or shoes, picking from the ash-barrels any little piece of half-burnt coal which by chance may be there. Half a basket is sometimes obtained from a single barrel; it is then taken to some desolate home to warm a failing mother or thriftless household. In view of this, is it not "mean" to make the servants riddle the ashes so that a single lump of coal might not be thrown out? and would it not be generous, even to put in the ash-barrel a handful or two of the best coal in order to reward these poor creatures? No!

Chance living seldom fails to end in moral depravities of the deepest dye, and in this very plain way. A person "finds" a plenty to day, and to-morrow, and may be for weeks together, and the feeling grows: "I will get a plenty more, and it is not worth while to be careful." But a night comes during which there is a storm of sleet or hail or deep drifting snow, and the ash-barrels are not put out, or by some other chance no coal is found, and the dreadful alternative is to freeze or obtain supplies of fuel in some other way. First comes the borrow, then the beg, and last the steal, for these three characteristics generally follow close on the heels of each other.

*Apropos*—In passing sixty-three East Sixteenth street, near Third Avenue, a few days since, one of our citizens, known for his activity and energy, and for his keen appreciation of the useful, said to us: "Buy one of my coal-sifters, cost sixteen hundred dollars to get out the patent, and I will sell you one for only four dollars and a half. I'll send it to your house, you shall keep it and try it, and if it does not meet your expectations, I will call for it." He sent it, called again, and got his——money. We now say to our readers, without the knowledge, consent, assent, or acquiescence of the gentleman aforesaid, that Booth's Patent Coal Ash-Sifter is the most perfect thing of the kind ever yet presented to the public. You empty the scuttle into the top of the sifter without any more dust than would arise from throwing it into the ash-barrel, and without turning any crank, or shaking, or any thing else on your part, there is a perfect separation of ash and coal by the time the empty scuttle is set down on the floor, without further dust, there not being found in the ashes a bit of coal as large as a common thimble, nor among the coal a half a pint of ashes, the ashes in one box, the coal in the other, all in an almost air-tight wooden inclosure, hence the suppression of dust.

